# Population re‐establishment and spatial dynamics of crowberry (*Empetrum nigrum* ssp. *hermaphroditum*), a foundation species in restored alpine ecosystems

**DOI:** 10.1002/ece3.70242

**Published:** 2024-09-12

**Authors:** Jan Sulavik, Inger Auestad, Stéphane Boudreau, Rune Halvorsen, Knut Rydgren

**Affiliations:** ^1^ Department of Civil Engineering and Environmental Sciences, Faculty of Technology, Environmental and Social Sciences Western Norway University of Applied Sciences Sogndal Norway; ^2^ Geo‐Ecological Research Group, Section for Research and Collections, Natural History Museum University of Oslo Oslo Norway; ^3^ Division for Energy, Environmental and Transport Statistics, Department for Business and Environmental Statistics Statistics Norway Kongsvinger Norway; ^4^ Département de Biologie et Centre d'Etudes Nordiques Université Laval Québec City Quebec Canada

**Keywords:** alpine, *Empetrum nigrum* ssp. *hermaphroditum*, population, restoration, spatial

## Abstract

Many ecosystems are defined and shaped by one or a few common, foundation species. Even though such species hold a key role in the restoration of these ecosystems, the demographic processes involved in their re‐establishment have rarely been studied. Foundation species' population dynamics, re‐establishment history, and the abiotic and biotic factors that affect individual establishment at restored sites can be studied by addressing population spatial patterns and age structure. Such an approach to studying population dynamics is particularly relevant for long‐lived species with low mortality, such as shrubs in alpine areas. We studied a population of the foundation species *Empetrum nigrum* ssp. *hermaphroditum* at an alpine spoil heap site and found evidence of population re‐establishment starting within a decade after construction. High *Empetrum* densities close to the spoil heap edges indicated that short distances to seed sources in the surroundings had a strong positive effect on establishment of individuals. *Empetrum* individuals were significantly clustered, which indicated intraspecific facilitation. As revealed by spatial analyses of recruits and older, established individuals, clustering developed gradually over time, which indicated a shift from no interaction to increased facilitation. We conclude that intraspecific facilitation promotes *Empetrum* reestablishment at the studied alpine spoil heap. Synthesis: We show that population spatial patterns and age structure can be successfully used to unveil the re‐establishment history of a foundation species in a restoration context. Efficient seed dispersal and intraspecific facilitation seem to be important factors behind *Empetrum*'s successful re‐establishment at alpine spoil heaps. Identification of abiotic and biotic factors determining foundation species' establishment success at restored sites can support planning and improve success of restoration.

## Graphical abstract

We present a hindcast of population dynamics of a foundation species (*Empetrum nigrum* ssp. *hermaphroditum*) at a restored alpine site based on individual spatial patterns and ages. This hindcast suggests that efficient seed dispersal and intraspecific facilitation are important factors behind successful population re‐establishment. We demonstrate that foundation species' population spatial pattern and age structure can be used to identify abiotic and biotic factors determining restoration success.

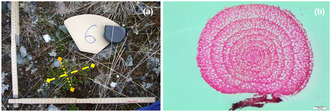

## 1 INTRODUCTION

Ecological restoration is becoming increasingly important in a world with a steadily growing human population and increasing land degradation (Perring et al., 2015). Research in restoration ecology has focused mainly on the ecosystem and community levels (Brudvig, 2011; Romanelli et al., 2018), while the population level has received much less attention (Harzé et al., 2018; Shriver et al., 2019). This is unfortunate because many ecosystems are defined by one or a few foundation species, that is abundant species that dominate the community, determine the diversity and modulate the energy and nutrient fluxes in the ecosystem (Ellison, 2019). The population dynamics of foundation species can significantly influence ecosystem recovery (Shriver et al., 2019) and early colonisers can impact strongly on community assembly through priority effects (Weidlich et al., 2021). Population‐level studies are therefore essential to provide insights into the mechanisms involved in recovery processes (Montalvo et al., 1997).

Plant population dynamics are typically studied by detailed demographic surveys (Colas et al., 2008), which, however, require long‐term data series (Shriver et al., 2019). Moreover, the long‐term, asymptotic dynamics inferred from repeated surveys may differ from the short‐term, transient dynamics (Iles et al., 2016) that are particularly important in restoration contexts (Shriver et al., 2019). Population dynamics can, however, also be studied retrospectively by deduction from spatial patterns of individuals and size and age structure of populations (Boudreau et al., 2010; Wiegand et al., 2000). These properties are shaped by recruitment and mortality histories (Hutchings, 1997) and provide a synthesis of population dynamics over a longer period (Wiegand et al., 2000). This approach is particularly useful for long‐lived species of trees and shrubs (Cousins et al., 2014) and typical foundation species (Ellison, 2019).

Plant spatial patterns reflect underlying ecological processes (Jeltsch et al., 1999; Law et al., 2009; Wiegand et al., 2003) and can reveal the relative contribution of different abiotic and biotic factors to plant establishment success (Fan, 2018; Lutz et al., 2014; Rice et al., 2012). Spatial patterns can also reveal otherwise unobtainable characteristics of temporal dynamics (Wiegand et al., 2003) that are reflected in population age structure (Crawley, 1990) and can influence recovery (Suding, 2011). Furthermore, spatial patterns can reveal interactions between plants in many ecosystems, including the alpine (Kikvidze et al., 2005). Accordingly, analyses of the spatial patterns of foundation species at restored sites may provide important insights into recovery processes. Such an approach to studying population dynamics has, however, rarely been used in a restoration context (e.g. Hein et al., 2020).

In alpine ecosystems, restoration is challenging due to generally slow vegetation establishment (Rydgren et al., 2013, 2020), although establishment rates of different species vary (Rydgren et al., 2013). Many alpine species have relatively short dispersal distances (Urbanska et al., 1998). Proximity to seed sources therefore enhances plant establishment at restored sites (Novák & Konvička, 2006). Nonetheless, we know little about how distance to seed sources affects the population dynamics of rapidly establishing species at restored alpine sites. Biotic interactions, either positive (i.e. facilitation) or negative (i.e. competition), can also affect plant establishment and thus the outcome of restoration (McCallum et al., 2018). However, interactions between plants are not necessarily static, but may shift in intensity and from positive to negative or vice versa over time (Anthelme et al., 2017; le Roux et al., 2013; Soliveres et al., 2010). Such shifts have been little studied in alpine ecosystems (Anthelme et al., 2017; Anthelme & Dangles, 2012) and only occasionally in a restoration context (e.g. Liu et al., 2020).

Facilitation usually dominates over competition (Callaway et al., 2002), and the presence of nurse plants can enhance restoration success in alpine ecosystems (Padilla & Pugnaire, 2006). Within a population, older individuals can act as nurse plants and facilitate the establishment of new conspecifics (Eränen & Kozlov, 2008). Alpine dwarf shrubs may act both as nurse plants and foundation species (Ballantyne & Pickering, 2015; Cáceres et al., 2015), but most dwarf‐shrub species, such as *Vaccinium* spp., tend to establish in restored alpine sites at later successional stages (Hagen, 2002; Rydgren et al., 2020). Crowberry (*Empetrum nigrum* ssp. *hermaphroditum*; hereafter–*Empetrum*), however, is an exception to this pattern, tending to establish in the early phase of restoration (Rydgren et al., 2020).

*Empetrum* is an evergreen dwarf shrub and a foundation species in Arctic and alpine ecosystems in the Northern Hemisphere (Bråthen et al., 2018; Bråthen & Ravolainen, 2015). *Empetrum* is monoecious and produces abundant fruits (drupes) with multiple seeds per fruit (Bienau et al., 2014). The seeds are dispersed by birds and other animals (Bell & Tallis, 1973), but also by wind (Ryvarden, 1971). Although often considered to reproduce mainly vegetatively (Bell & Tallis, 1973), *Empetrum* can also efficiently reproduce sexually (Bienau et al., 2016; Boudreau et al., 2010). Rapid establishment was observed in some disturbed Arctic sites (Angers‐Blondin & Boudreau, 2017) and restored alpine spoil heaps, that is piles of surplus rock from hydropower construction (Rydgren et al., 2013). However, we know little about how distance to seed source affects the establishment and population dynamics of *Empetrum* in restored alpine ecosystems. In the absence of disturbance, the mortality of *Empetrum* is usually low (Angers‐Blondin & Boudreau, 2017; Hill et al., 2012), and *Empetrum* individuals can interact positively as well as negatively with other species (Cutler et al., 2008b; Pellissier et al., 2010). While it can act as a nurse plant for other species, for example, *Carex bigelowii* (Carlsson & Callaghan, 1991), it can also inhibit seed germination (González et al., 2014) and reduce species richness at a site (Bråthen et al., 2018; Bråthen & Ravolainen, 2015). However, little is known about the influence of intraspecific interactions between *Empetrum* individuals on the recovery process at restored alpine sites.

Here, we explore how abiotic factors, particularly local spatial and topographic conditions determining distance to seed sources, and biotic factors, particularly intraspecific interactions, affect the population dynamics and establishment success of *Empetrum* in a restored alpine ecosystem. We utilise *Empetrum's* population spatial pattern and deduce population age structure from the allometric relationship between individual size and age (Boudreau et al., 2010). Based on the spatial pattern and ages of individuals, we aim to fulfil the following objectives:

1. to reconstruct the re‐establishment of the *Empetrum* population at an alpine spoil heap site
2. to assess how distance to seed sources affects *Empetrum* establishment, and
3. to study potential intraspecific interactions in *Empetrum*, particularly between recruits and older, established individuals and eventual interaction shifts over time.

Hypothetically, *Empetrum* re‐establishment at our study site can either (a) progress from the edges as a “colonisation front” (cf. Boudreau et al., 2010) or (b) be spread homogeneously across the site because of its relatively small diameter and efficient animal/wind dispersal. We would like to find out if we can detect spatial patterns consistent with hypotheses (a) or (b) as preliminary investigations were inconclusive.

Fulfilling these objectives can improve our understanding of a foundation species' establishment success in a restoration context.

## 2 MATERIALS AND METHODS

### 2.1 Study area

The study was carried out at the alpine spoil heap at Fossane, Aurland municipality, Western Norway (60° 47.292′ N, 7° 23.821′ E, ca. 1270 m a.s.l.), constructed in 1984. It covers 41,000 m^2^ and consists of coarse‐grained blasted rocks, mainly gneiss with some phyllite (Skjerdal & Odland, 1995). The topmost layer has little microtopographic variation. Shortly after construction, the spoil heap was fertilised and seeded with a seed mixture that probably contained commercial varieties of *Festuca ovina*, *F. rubra*, *Agrostis capillaris*, *Phleum pratense* and *Schedonorus pratensis* (Skjerdal & Odland, 1995). The spoil heap has been regularly grazed by sheep at low densities. The Fossane spoil heap consists of a northern, a southern and an eastern section, which are separated by a road and a brooklet (Skjerdal & Odland, 1995). The eastern part contains an unvegetated parking lot that was not included in our study (Figure S1).

The study site is located on a west‐facing valley slope. The estimated average annual temperature and precipitation at Fossane over the period 1971–2000 were −1.95°C (NVE, 2021b) and 1855 mm (NVE, 2018), respectively. On average, snow cover >5 cm was present 265 days per year (NVE, 2021a). The site is situated in the clearly oceanic bioclimatic section and the upper part of the low alpine bioclimatic zone (Moen, 1999).

The study site is surrounded by dwarf‐shrub heaths dominated by *Vaccinium uliginosum*, *V*. *myrtillus*, *V*. *vitis‐idaea* and *Salix herbacea* with scattered groups of the larger shrubs *Salix glauca* and *S*. *lapponum*. Such vegetation is typical of the upper part of the low alpine zone in Norway (Rydgren et al., 2013). *Empetrum* is abundantly present around the entire spoil heap (Rydgren et al., 2020). Limited vegetative reproduction of *Empetrum* at the spoil heap allowed identification of individual plants, a prerequisite for analyses of establishment patterns (Boudreau et al., 2010).

### 2.2 Sampling and data preparation for allometric modelling

In September 2015, at the end of the growing season, we established six transects across the spoil heap from west to east: four in the southern section and two in the northern section. The distance between transects was ca. 30 m (Figure S1). In these so‐called “allometric transects,” each 6 m wide and 30 m long, we selectively collected 90 *Empetrum* individuals with principal crown diameter (PCD) 3–120 cm to determine the allometric relationship between size and age. The sampled range of PCD was based on a preliminary survey of individual sizes in the area. An individual was distinguished by having a single root collar and a single crown.

**FIGURE 1:**
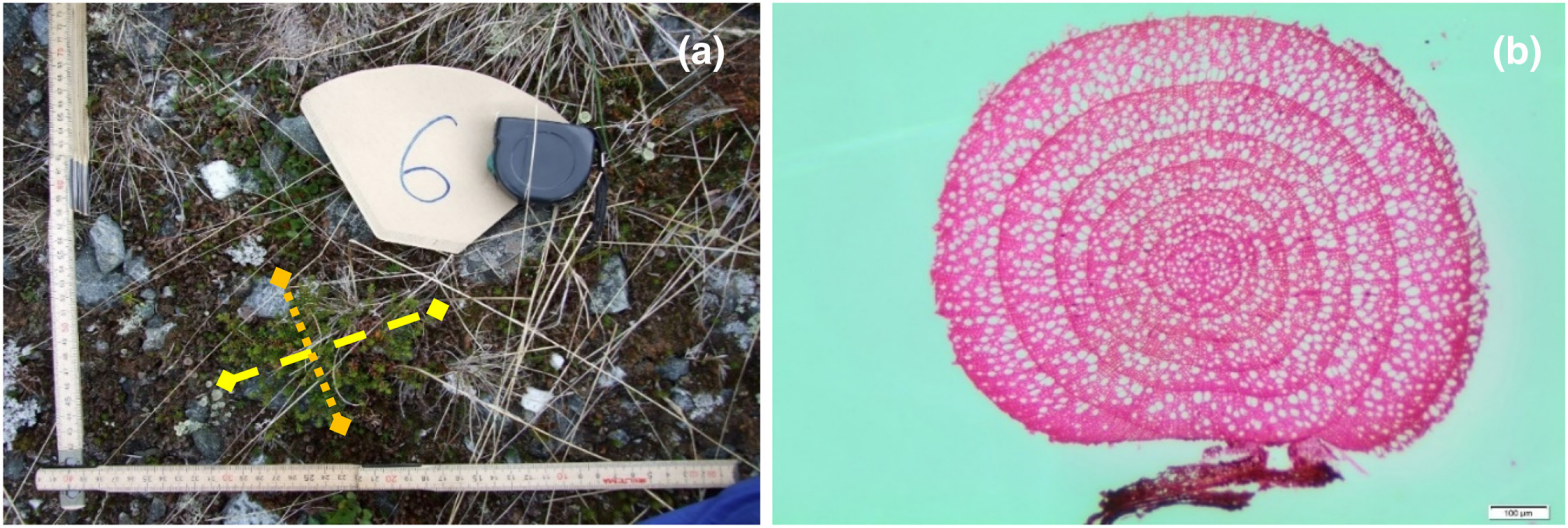
*Empetrum* sampling and preparation for allometric modelling. (a) *Empetrum* individual ID6 with principal crown diameter (PCD, yellow dashed line) 15 cm and largest diameter perpendicular to PCD (LPD, orange dotted line) 11 cm. (b) Digital image of root collar cross‐section of ID6 with minimum age estimated to 8 years, based on growth‐ring count.

We photographed (with a scale) all *Empetrum* individuals in the field and measured PCD (the largest crown diameter) and LPD (the largest crown diameter perpendicular to PCD) in 60 of the 90 individuals before collection (Figure 1a). As all individuals were completely prostrate, that is the crown area of an individual was identical with the crown projection on the soil surface, we measured the crown diameters along the surface. Adverse weather precluded field measurements of the remaining 30 individuals. Their PCD and LPD were instead measured on the photographs using ImageJ (Schneider et al., 2012). PCD and LPD were used to represent the size of *Empetrum* individuals. All collected individuals were mapped using GNSS survey equipment with a stated precision of <1 cm.

We determined the age of all collected *Empetrum* individuals by the method of Boudreau et al. (2010): Root collars were air‐dried at ambient room temperature (ca. 21°C) for ca. 2 months, boiled in water for at least 2 h, mounted to a sledge microtome and sliced to 20 μm cross‐sections that were stained with a safranin solution. Stained cross‐sections were stabilised in ethanol, dried and mounted on glass slides by a low‐viscosity medium. We counted growth rings on digital images obtained from a dissecting microscope (Figure 1b). The number of growth rings was recorded as the individuals' minimum age.

Upon close laboratory examination, three of the 90 collected plants were found to consist of two intertwined individuals each. These were discarded. Size and age data for the remaining 87 individuals made up the allometric dataset that we used to build an allometric, that is size‐for‐age, model for *Empetrum*.

### 2.3 Sampling for reconstruction of population re‐establishment

In July 2016, at the beginning of the growing season, we selected three “establishment transects” (hereafter: transects 1–3), each 5 m wide and running from west to east across the northern section of the spoil heap (Figure S1). Transect 1 followed the northern spoil heap edge, while transects 2 and 3 were extensions of two “allometric transects.” Transect lengths ranged from 55 to 100 m. All transects faced west and were gently sloping, with an average inclination of ca. 20°. Transects had generally little topographic variation, apart from transect 2, which crossed a mound close to the eastern spoil heap edge (Figure S2). Before recording the *Empetrum* individuals, each transect was divided into contiguous 5 × 5 m segments. Three segments were omitted from analyses: The two most westerly segments of transect 1 that were devoid of *Empetrum*, and one segment in transect 3 from which data were lost because of equipment malfunction (Figure S1).

We photographed (with a scale) all *Empetrum* individuals, measured their size (PCD and LPD) and mapped their positions by GNSS survey equipment. We noted when an individual was senescent, that is had discoloured, withered or missing leaves. The 2251 *Empetrum* individuals made up the establishment dataset (also including 28 individuals collected for the allometric dataset), which was used to reconstruct the re‐establishment history of *Empetrum* at the study site (for details, see Appendix S1 and Table S1).

### 2.4 Allometric modelling

We used R version 3.4.2 (R Core Team, 2015) for all analyses. We build an allometric model for *Empetrum* based on the allometric dataset. GLMs with Poisson errors were obtained, with age as the response variable and log‐transformed size measurements (PCD and LPD) as predictors (for details of allometric modelling, see Appendix S2). PCD and LCD were strongly correlated (Kendall's rank correlation coefficient *τ* = 0.87, *p* < .001, *n* = 87). Accordingly, the best GLM model was: Age = exp(1.28 + 0.27*log_2_(PCD)), with pseudo‐*R* ^2^ = 0.73 (*p* < .001, n = 87; see Figure S3 and Table S2). The average absolute‐value difference between observed and modelled age was 1.9 years for all individuals (*n* = 87), and 2.7 years for individuals with observed age <6 years (*n* = 8).

We used the modelled allometric relationship to estimate the age of all *Empetrum* individuals in the establishment dataset with a precision of 0.1 years. As sampling was conducted between two growing seasons, all individual age estimates are expressed as of 1 January 2016.

### 2.5 Analyses for reconstruction of population re‐establishment

We used ArcGIS (ESRI, R, 2011) to prepare spatial data and the R package “spatstat” (Baddeley et al., 2015) for all spatial analyses (for details, see Appendix S3 and Appendix S4). We treated the mapped positions of all *Empetrum* individuals in the establishment dataset as a spatial point pattern. As *Empetrum* was present in the undisturbed surroundings along the entire spoil heap edge, we used the distance to the nearest edge as a proxy for the distance to the nearest seed source.

We used the spatial pattern and age structure to reconstruct the establishment history of *Empetrum* at the study site. We observed no dead individuals during sampling and hence assumed zero mortality in analyses (Boudreau et al., 2010). We divided *Empetrum* individuals into seven age classes, numbered from 1 to 7 (Table 1). Age class 1 comprised the oldest individuals (estimated age >21 years at the time of sampling), age classes 2–6 covered five consecutive three‐year intervals and age class 7 comprised the youngest individuals with estimated age <6 years. Age class 7 was kept broad because of more uncertain age estimates for the youngest plants. Age classes were non‐overlapping: for example, an individual with an estimated age of 21.1 years or higher at the time of sampling would be placed in age class 1, an individual with an estimated age between 18.1 and 21.0 in age class 2, etc. We chose to use seven age classes to cover the age span between the oldest and the youngest individuals (>20 years) and show changes over time on a relatively fine scale, but simultaneously keep the number of the age classes sensible.

**TABLE 1:** *Empetrum* population (established individuals and recruits; age classes, ages and *n*) in transects 1–3 at six time points, yac = years after the construction of the spoil heap.

Time point	Established individuals			Recruits		
Yac	Age class	Ages	*n*	Age class	Ages	*n*
13	1	>21 (21–24)	22	2	18–21	89
16	1–2	18–24	111	3	15–18	254
19	1–3	15–24	365	4	12–15	432
22	1–4	12–24	797	5	9–12	512
25	1–5	9–24	1309	6	6–9	565
31	1–6	6–24	1874	7	<6 (1–6)	377

*Note*: The last time point (yac = 31) is the time of sampling.

Variations in *Empetrum* densities between and within transects, calculated separately for the seven age classes, were analysed for the assessment of the effect of distance to seed source on the establishment. We also obtained a smoothed estimate (Baddeley et al., 2015) of *Empetrum* density at the time of sampling as a function of distance to the nearest spoil heap edge.

We used the pair correlation function (PCF) to study spatial relationships between *Empetrum* individuals, that is clustering or dispersion, at the time of sampling. In alpine areas, clustering suggests facilitation while dispersion suggests competition between plants (Kikvidze et al., 2005). By PCF, the observed frequency of between‐point (in our case between‐individual) distances is compared with the frequencies expected under a null model of no relationship (Baddeley et al., 2015). Higher observed PCF value than predicted by the null model at a given distance indicates clustering while lower value indicates dispersion (Baddeley et al., 2015). Unlike the commonly used Ripley's K function, PCF is non‐cumulative and allows for precise assessment of clustering or dispersion at specific distances (Velázquez et al., 2016). We used univariate inhomogeneous PCF (uiPCF) to account for variation in *Empetrum* density within transects. We specified the null model as an inhomogeneous Poisson process and compared observed uiPCF values to values obtained from 78 simulations of the null model: 39 to estimate the mean and 39 to obtain 95% confidence intervals (global envelopes) around the mean (Baddeley et al., 2014).

We addressed the chronology of population re‐establishment and possible interaction shifts by studying spatial relationships of *Empetrum* at six different time points between the construction of the spoil heap and the time of sampling. Time points are referred to by years after construction (yac), the last time point (yac = 31) being the time of sampling (Table 1). At each time point, the youngest *Empetrum* individuals (i.e. the most recent age class relative to the time point) were treated as recruits, while older individuals (i.e. earlier age class(es) relative to the recruits) were treated as established, potential nurse plants (Table 1).

We used bivariate inhomogeneous PCF (biPCF) to study spatial relationships between *Empetrum* recruits and established individuals at each time point. The null model, specified as an inhomogeneous multitype Poisson process, was that recruits had no significant spatial relationship with established individuals. For each time point, we compared observed biPCF values to values obtained from 78 null model simulations. Deviations from null models indicated significant clustering or dispersion of recruits around established individuals at a given time point.

All PCFs were evaluated graphically and subjected to Diggle–Cressie–Loosmore–Ford (DCLF) tests (Baddeley et al., 2014).

## 3 RESULTS

**FIGURE 2:**
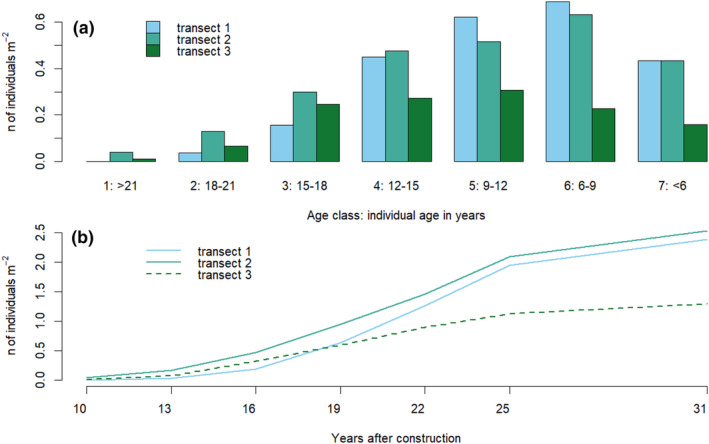
Age structure of *Empetrum* in transects 1–3. (a) Age structure expressed as the average density (i.e. number of individuals per age class per m^2^) of each of the seven age classes 1–7 that correspond to specific time intervals after construction of the spoil heap. (b) Cumulative average *Empetrum* density in the transects under the assumption of zero mortality.

Re‐establishment of the *Empetrum* population started within 10 years after the spoil heap construction in 1984, that is more than 20 years before the sampling. The individual with the highest estimated age, 23.9 years, was recorded in transect 2. According to the allometric model, this individual established in 1991, that is 7 years after construction (yac). By 1994 (yac = 10), individuals in age class 1 (>21 years old at the time of sampling) had established in transects 2 and 3 (Figure 2a). In transect 1, the highest estimated individual age was 20.6 years, suggesting establishment in 1995. The age structure of the *Empetrum* population differed among transects (Figure 2a). In transect 1, establishment occurred at lower rates than in the other transects till ca. yac = 16, peaked ca. yac = 25 and then declined. Transect 2 followed the same pattern except for the higher establishment rates yac = 7–16. In transect 3, establishment rates reached peak levels ca. yac = 22 and declined afterwards.

At the time of sampling (yac = 31), *Empetrum* densities were similar in transects 1 and 2 (2.4 and 2.5 individuals per m^2^, respectively, Figure 2b) and almost twice as high as in transect 3 (1.3 individuals per m^2^). The size structure of *Empetrum* followed a J‐shaped curve in all three transects (Figure S4). Transect 1 had the highest proportion of senescent individuals (28 out of 721; 4%), followed by transect 2 (23 out of 1180; 2%) and transect 3 (0 out of 350; 0%).

**FIGURE 3:**
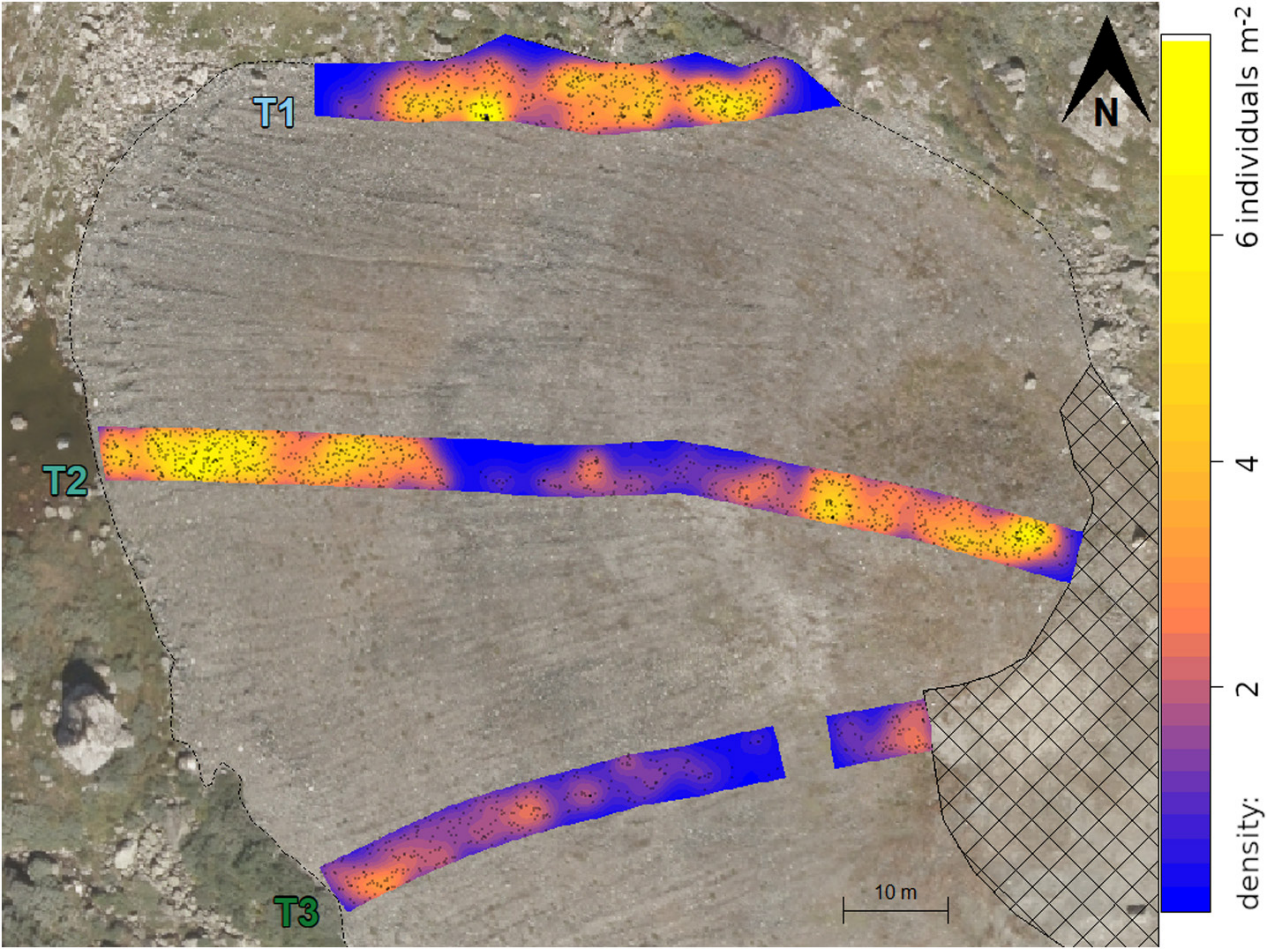
Spatial patterns and densities of *Empetrum* individuals in transects 1–3 (T1–T3). Points = *Empetrum* individuals in the establishment dataset (*n* = 2251). Colours = densities, that is number of individuals per m^2^. Cross‐hatched area = unvegetated parking lot. Dash‐dotted line = spoil heap edge.

At the time of sampling, *Empetrum* densities varied considerably within the three transects. The highest *Empetrum* densities were found close to, but not directly at, the spoil heap edges, while the lowest densities were found near the centres of transects 2 and 3 (Figure 3). At the mound in the eastern part of transect 2, somewhat lower densities were observed on the top compared with the slopes of the mound (Figure 3, see also Figure S2a).

**FIGURE 4:**
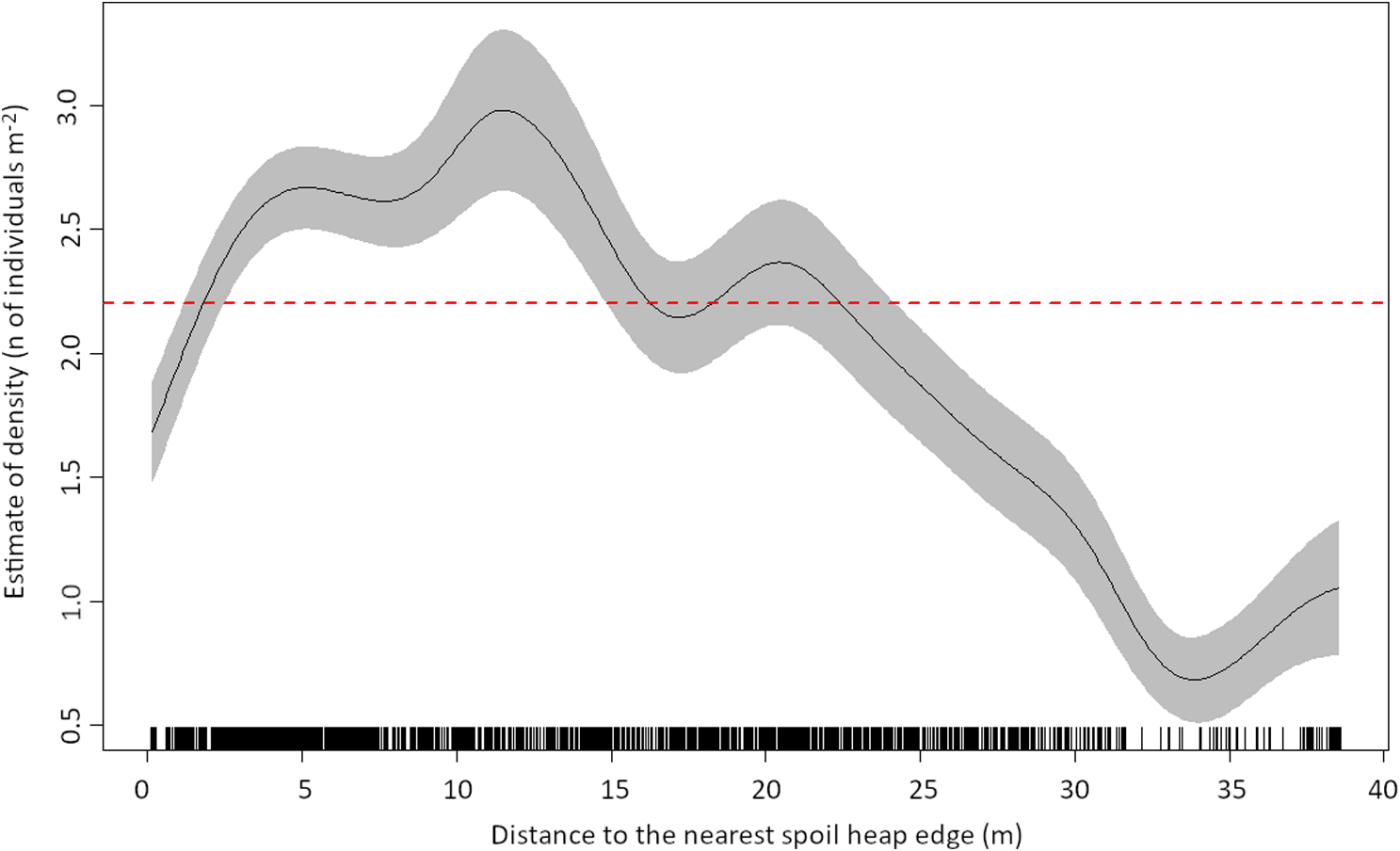
Estimate of *Empetrum* density as a function of distance to the nearest spoil heap edge, based on the establishment dataset (*n* = 2251). Solid black line = mean estimated *Empetrum* density. Grey band = 95% CI of the estimate. Tick marks above the x‐axis = a rug plot of observed distances of *Empetrum* individuals to the nearest spoil heap edge. Dashed red line = overall average *Empetrum* density in transects 1–3 (2.2 individuals per m^2^).

The density of *Empetrum* across transects, quantified by the smoothed estimate, was strongly related to the distance to the nearest spoil heap edge (Figure 4), peaking at ca. 3 individuals per m^2^, 11.5 m from the nearest edge. Above‐average densities were estimated at 1.8–22.5 m from the nearest edge.

**FIGURE 5:**
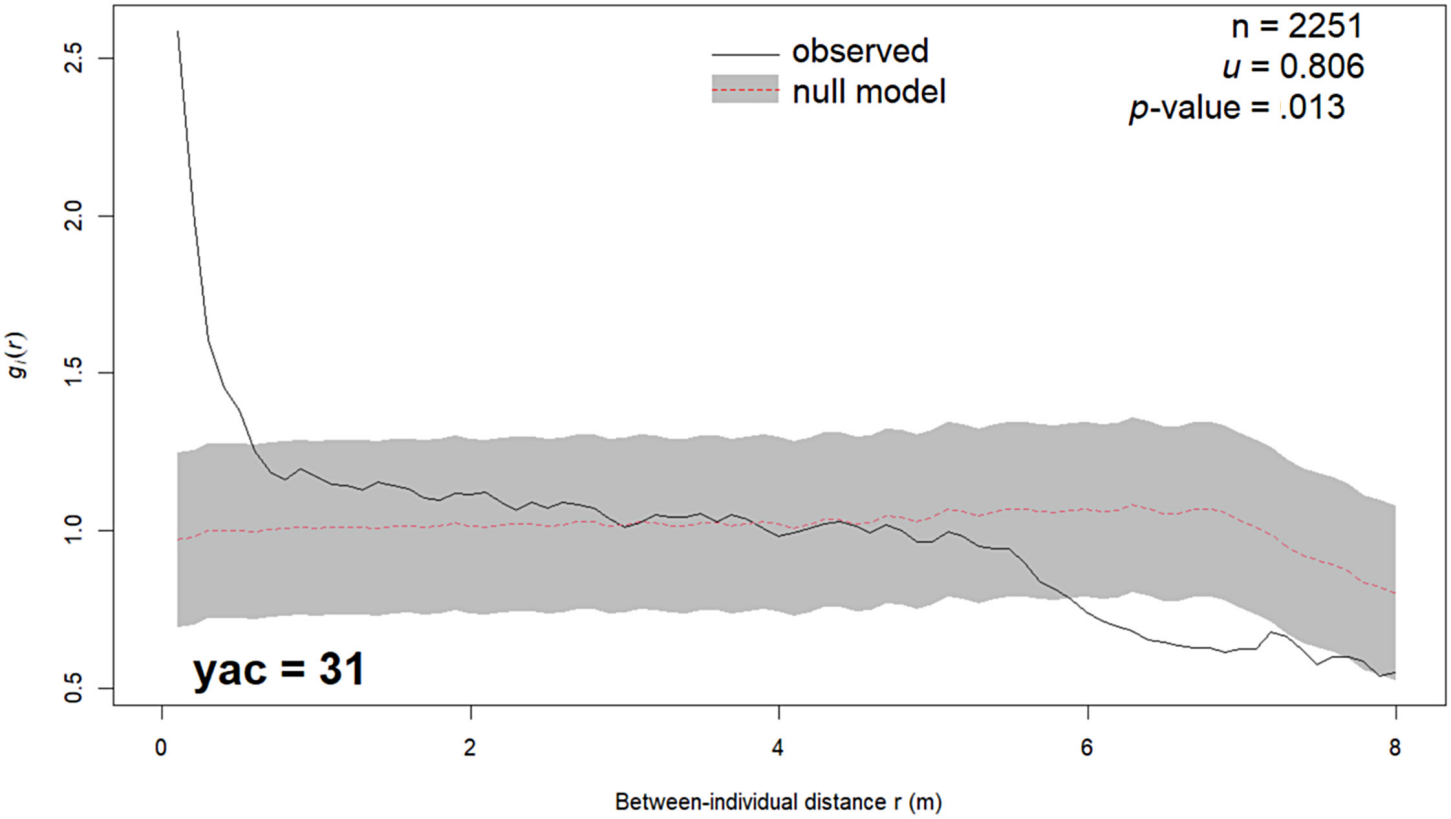
Spatial relationships between *Empetrum* individuals in the establishment dataset (*n* = 2251) at the time of sampling (yac = 31), assessed by the univariate inhomogeneous pair correlation function (uiPCF). Observed values of uiPCF, g_i_, above/below the global envelope indicate significant clustering/dispersion of individuals. Solid black line = observed g_i_ values at between‐individual distance r. Grey band = the global envelope, 95% CI of g_i_ values from the null model simulations. Dashed red line = mean simulated g_i_ values. *U*‐ and *p*‐values from the two‐tailed Diggle–Cressie–Loosmore–Ford (DCLF) test.

**FIGURE 6:**
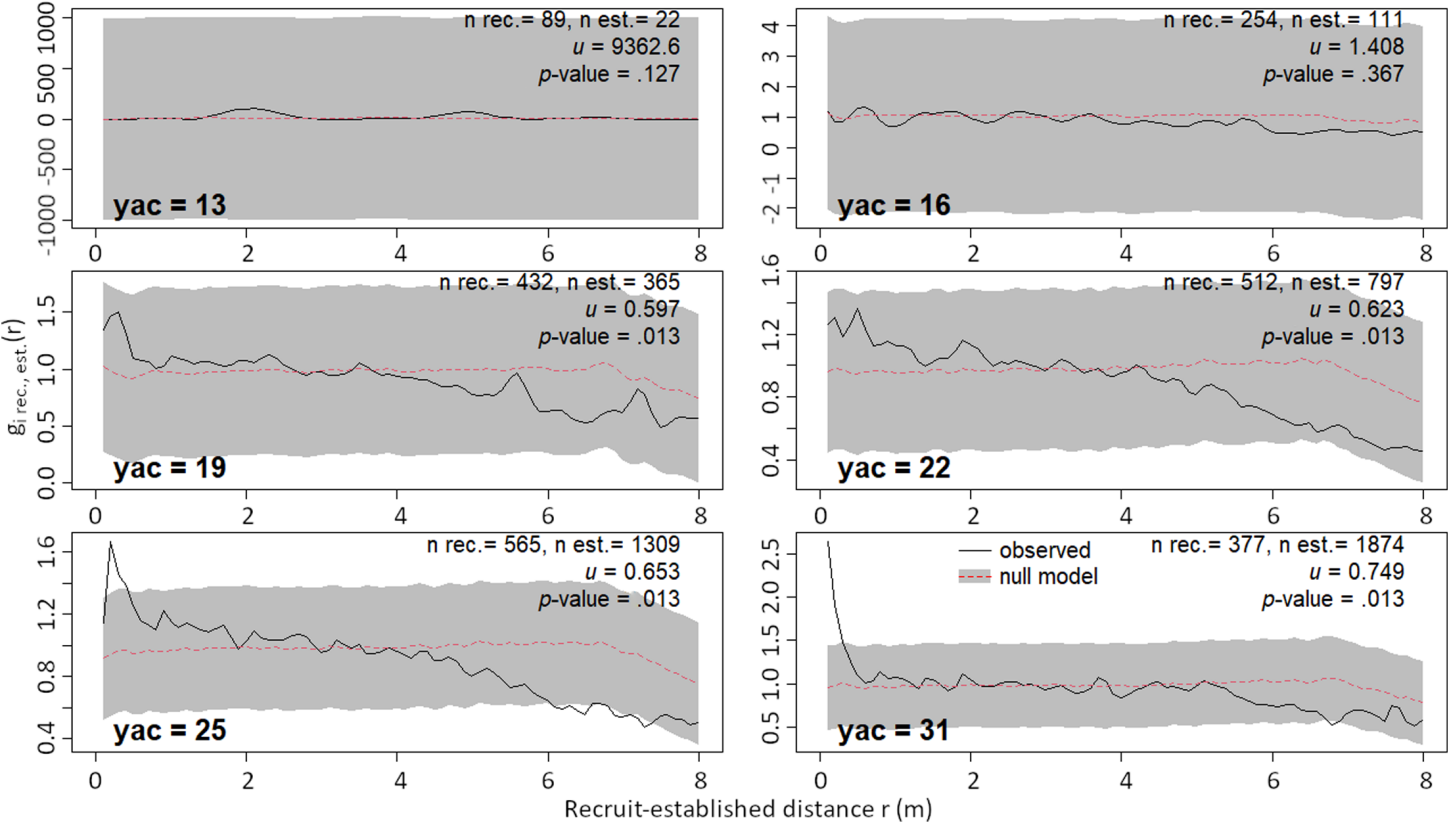
Spatial relationships between *Empetrum* recruits and established individuals at six time points (see Table 1), assessed by bivariate inhomogeneous pair correlation functions (biPCF). Observed values of biPCF, g_i,rec,est_ above/below the global envelope indicate significant clustering/dispersion of recruits around established individuals. n rec. = number of recruits, n est. = number of established individuals. For explanation of symbols, see Figure 5.

At the time of sampling, *Empetrum* showed a non‐random spatial pattern: Individuals were more often found close to each other (<0.5 m), that is clustered, than what would be expected at random (Figure 5). In addition, individuals were less often found at larger distances from each other (>5.9 m), that is dispersed, than what would be expected at random. The significance of the spatial relationship between all *Empetrum* individuals was confirmed by the graphical evaluation of observed vs. simulated null model PCF values and by the DCLF test (*u* = 0.806, i‐value = .013). This spatial pattern developed gradually (Figure 6). In the early phase of *Empetrum* population re‐establishment until yac = 16, recruits were randomly distributed around established individuals. Thereafter, recruits gradually clustered close to established individuals until this pattern had become significant at yac = 25. The DCLF tests (*u*‐ and *p*‐values in Figure 6) did, however, indicated a significant clustering of recruits close to established individuals already at yac = 19.

## 4 DISCUSSION

Our results show that the re‐establishment of the *Empetrum* population started 7–10 years after the construction of the studied alpine spoil heap and thereafter proceeded at increasingly higher rates for the next 10–15 years before establishment rates started to decline. This shows that the foundation species *Empetrum* can establish efficiently from seeds in restored alpine ecosystems. Furthermore, the observed spatial pattern of *Empetrum* individuals suggests that distance to seed sources strongly influences establishment. The observed patterns also suggest that facilitation between *Empetrum* individuals dominates over competition and that facilitation may gradually increase over time. We can therefore discard hypothesis (b), that is homogeneous *Empetrum* establishment across the spoil heap. However, the establishment patterns did not form a distinct “colonisation front,” and we thus do not see conclusive evidence for hypothesis (a) either.

### 4.1 Re‐establishment history and effects of distance to seed sources

General patterns of variation in *Empetrum* density across our study site indicate that the distance to seed sources determines the establishment of *Empetrum* individuals. This accords with the results of Boudreau et al. (2010) who studied the establishment of *Empetrum* at sub‐Arctic sand dunes. Higher *Empetrum* density closer to the spoil heap edge than near the centre indicates efficient seed dispersal from the surroundings to the spoil heap. However, *Empetrum* densities were lower than the overall average in the immediate vicinity (≤1.8 m) of the spoil heap edge. This zone is characterised by relatively coarse material, unfavourable for re‐establishment of most species (Rydgren et al., 2013).

Furthermore, our results suggest that seed dispersal from *Empetrum* individuals growing on the spoil heap contributes to population growth in later phases of re‐establishment. Observations of ripe drupes on individuals with principal crown diameter down to ca. 13 cm, which corresponds to an estimated individual age of ca. 10 years (unpublished data), accords with the observed peak recruitment around 20 years after spoil heap construction. Observations in the eastern part of transect 2 indicate that *Empetrum* establishment is also influenced by local topography (i.e. convex, or concave terrain): *Empetrum* densities at the mound top are somewhat lower than at the adjacent slopes and lower than expected from its distance to the nearest spoil heap edge. Similar observations are reported by Angers‐Blondin and Boudreau (2017), who found consistently lower *Empetrum* densities on dune ridges than on depressions between the ridges, regardless of the distance to seed sources.

The higher average density and different age structure indicate higher rates of *Empetrum* establishment in transects 1 and 2 than in transect 3, as expected from the longer average distance of the latter to seed sources. However, the relative scarcity of the youngest individuals (age class 7; <6 years at the time of sampling) in all three transects is surprising. Temperatures and precipitation have a strong impact on seed production in *Empetrum* (Bienau et al., 2014), which may have influenced recruitment in years prior to the sampling. In addition, young *Empetrum* individuals can quickly reach large sizes due to warming (Angers‐Blondin & Boudreau, 2017), which also can result in fewer small *Empetrum* individuals than expected from the previous population development (Boudreau et al., 2010). Growth in *Empetrum* is also strongly linked to microsite conditions, particularly soil moisture and snow cover and duration (Dobbert et al., 2021). Lastly, the precision of the allometric model may have played a role. The modelled population age structure reflects the chronology of *Empetrum* population re‐establishment, but allometric models may be improved by including more measured individual parameters, such as stem diameter (Hegland et al., 2010).

The higher average density of *Empetrum* in transect 2 than in transect 1 situated along the spoil heap edge close to seed sources most likely reflects environmental differences between the two transects, particularly concerning snow depth. Transect 1 lies beneath a steep slope where snow is likely to accumulate in winter. Long‐lasting snow cover has a strong effect on *Empetrum* growth and reproduction (Bienau et al., 2014), as reflected in the declining *Empetrum* abundances towards snow beds (Dahl, 1956). Long‐lasting snow cover is also known to increase the abundance of fungal pathogens such as the *Empetrum*‐specific fungus *Arwidssonia empetri* (Olofsson et al., 2011). Although we observed no dead *Empetrum* individuals in the transects, the higher proportion of senescent individuals observed in transect 1 compared to transects 2 and 3 supports this explanation.

*Empetrum* seeds are known to disperse over distances up to 200 m (Boudreau et al., 2010) and can most likely disperse even further. Nevertheless, our results suggest that successful restoration involving *Empetrum* would benefit from shorter distances between the centre of a restoration site and the seed sources. In accordance with Boudreau et al. (2010) and Angers‐Blondin and Boudreau (2017), our results suggest that terrain form should also be considered and, more specifically, that *Empetrum* establishment may be lower at convex terrain forms like ridges. Alpine ridges lack a stable snow cover and are susceptible to frost damage (Bokhorst et al., 2009), particularly at the onset of the growing season (Weijers et al., 2018). This affects growth in *Empetrum* and other dwarf shrubs negatively (Wipf et al., 2009). In addition, snow cover duration determines soil moisture vital for plant growth, and lacking snow cover can cause water shortage early in the growing season at the ridges (Bär et al., 2008). Varied topography with intermediate (i.e. neither long‐lasting nor unstable) snow cover and short dispersal distances may thus optimise the re‐establishment of *Empetrum* populations at alpine restoration sites.

### 4.2 Intraspecific interactions and interaction shifts

The significant clustering (i.e. more individuals than expected at random) at short between‐individual distances indicates a higher probability of *Empetrum* recruitment close to already established individuals. Similar clustering has been observed at lava flows (Cutler et al., 2008b) and sub‐Arctic sand dunes (Boudreau et al., 2010). Chronological analyses of spatial relationships between recruits and established individuals show that the spatial pattern of *Empetrum* on alpine spoil heaps develops gradually. In the early phase, individuals establish independently of each other by exploiting natural safe sites (Cutler et al., 2008a), while in the later phase, recruits gradually cluster close to the older, established individuals. A plausible explanation is that the older *Empetrum* individuals act as nurse plants or “niche constructors” (Odling‐Smee et al., 2013) for the recruits by creating new safe sites for further establishment (for thermal niches of alpine plants, see Löffler & Pape, 2020). This is noteworthy because *Empetrum* is generally considered an allelopathic species (Bråthen et al., 2010) as it produces phenolic compounds suppressing seedling establishment of other species (González et al., 2014). These compounds, however, are unlikely to accumulate at our study site as spoil heaps are well‐drained (Rydgren et al., 2013). Other possible explanations include very short‐range seed dispersal from mature individuals, but other evidence indicates frequent long‐range dispersal, and we lack precise data on dispersal kernel for *Empetrum*. At any rate, intraspecific facilitation seems to be an important biotic factor promoting *Empetrum*'s establishment at our study site.

The observed significant dispersion of individuals (i.e. fewer individuals than expected at random) at between‐individual distances above 5.9 m accords with the previously observed negative spatial autocorrelation between *Empetrum* patches at longer distances on lava flows (Cutler et al., 2008b). The mechanisms behind this pattern are unclear, as competition between plants is a neighbour phenomenon. Two potential explanations may apply, one ecological and one methodological. The ecological explanation is that in the later phase of re‐establishment, natural safe sites are occupied, and new safe sites for recruits are created by older, established individuals. This explanation simultaneously accounts for clustering at shorter and dispersion at longer distances. It further supports that intraspecific facilitation promotes *Empetrum* recruitment in later phases of population re‐establishment. The methodological explanation is that our 5‐m‐wide transects have caused an underrepresentation of longer between‐individual distances in the dataset. Mechanisms involved in both explanations may independently cause and/or enhance the observed pattern. At any rate, intraspecific competition does not seem to influence *Empetrum* population re‐establishment during the studied period.

Spatial patterns of foundation species are also very likely to influence the establishment of other species at restored sites (McCallum et al., 2018), particularly in the alpine zone (Gómez‐Aparicio, 2009; Padilla & Pugnaire, 2006). For example, the Australian alpine tundra shrub *Epacris gunii, a species with the same growth form and belonging to the same family (*i.e. *Ericaceae) as Empetrum*, acts as a nurse plant and facilitates the increase of species abundance and richness at multiple spatial scales (Ballantyne & Pickering, 2015). Interspecific interactions, however, are beyond the scope of our population‐level study.

## 5 CONCLUSIONS

Population dynamics and plant spatial patterns play an important role in restoration (Harzé et al., 2018; Shriver et al., 2019). Our *Empetrum* study is, to the best of our knowledge, the first where spatial patterns and age structure are used to study population dynamics of a foundation species in a restoration context. We demonstrate that new insights about abiotic and biotic factors affecting population re‐establishment at restored sites may be obtained from point pattern analyses of precise spatial data (Baddeley et al., 2015). We also show how non‐destructive estimation of individual ages by allometric models may provide valuable information about re‐establishment history. Our analyses of spatial and age data of *Empetrum* indicate that it can establish rapidly from seeds, but that dispersal distances matter. Moreover, spatial relationships between individuals indicate that established individuals facilitate the establishment of recruits. Efficient seed dispersal and intraspecific facilitation thus appear as important factors behind *Empetrum*'s successful re‐establishment on alpine spoil heaps and are likely to play a role in restoration success in other harsh environments. The spatially explicit approach to studying population dynamics which we used here can be relevant for many long‐lived foundation species in restored terrestrial ecosystems and should be explored further. Ultimately, a similar approach can also be used to assess interspecific interactions between foundation species such as *Empetrum* and others in future studies.

## AUTHOR CONTRIBUTIONS

**Jan Sulavik:** Conceptualization (lead); data curation (lead); formal analysis (lead); investigation (lead); methodology (equal); visualization (lead); writing – original draft (lead); writing – review and editing (lead). **Inger Auestad:** Funding acquisition (equal); project administration (supporting); supervision (supporting); writing – original draft (equal); writing – review and editing (equal). **Stéphane Boudreau:** Conceptualization (supporting); methodology (equal); supervision (supporting); writing – original draft (supporting); writing – review and editing (supporting). **Rune Halvorsen:** Conceptualization (equal); methodology (equal); supervision (equal); writing – original draft (equal); writing – review and editing (equal). **Knut Rydgren:** Conceptualization (equal); funding acquisition (lead); methodology (equal); project administration (lead); supervision (equal); writing – original draft (equal); writing – review and editing (equal).

## FUNDING INFORMATION

This study was funded by Sogn og Fjordane Energi, Sparebankstiftinga Sogn og Fjordane, Sogn og Fjordane Fylkeskommune, Norsk Hydro and the Research Council of Norway (project number 238281).

## CONFLICT OF INTEREST STATEMENT

No conflict of interest.

## Supporting information


Data S1.

## Data Availability

The data that support the findings of this study are openly available in Dryad at https://doi.org/10.5061/dryad.bzkh189k8.
