# Supplementary material for: Population re‐establishment and spatial dynamics of crowberry (Empetrum nigrum ssp. hermaphroditum), a foundation species in restored alpine ecosystems
Source: Ecol Evol. 2024 Sep 12;14(9):e70242. doi: 10.1002/ece3.70242 (PMC11392828; doi:10.1002/ece3.70242)
Supplement: Supplementary file 1 — Data S1. [file ECE3-14-e70242-s001.docx]

**APPENDIX: FIGURES**

Figure S1: Aerial image of Fossane spoil heap (main frame), detail of establishment transects (transects 1–3) in the northern spoil heap section (top right frame), and location of the study site within Norway (red point in the bottom left frame). Yellow points = *Empetrum* individuals in allometry dataset. Cross-hatched area = unvegetated parking lot. Dashed line = spoil-heap edge and sections (northern, southern, and eastern). White areas in the top right frame = transect segments omitted from analyses. Background orthophoto: Norge i bilder (Statens kartverk et al. 2020).


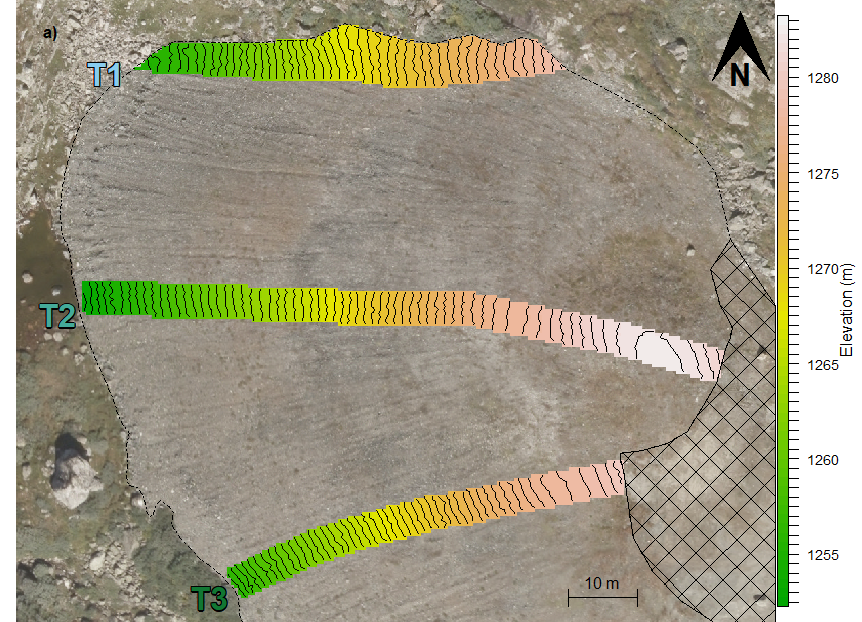

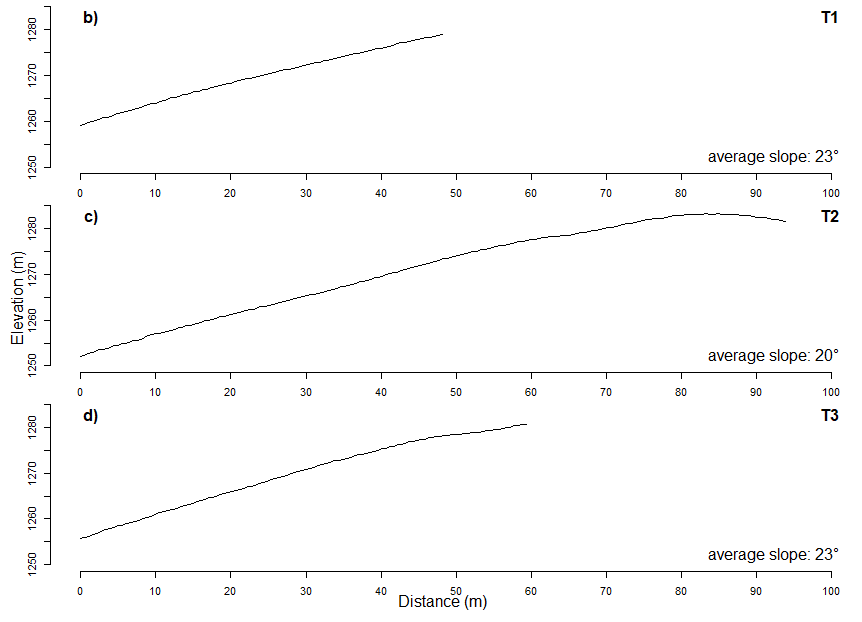


Figure S2: Topography and elevation profiles of transects 1–3 (T1–T3). a) Topography of T1–T3 based on digital surface model (Kartverket Bergen 2014). Contour (black lines) and colour intervals are 0.5 m. Cross-hatched area = unvegetated parking lot. Dash-dotted line = spoil-heap edge. Background orthophoto: Norge i bilder (Statens kartverk et al. 2020). b) Elevation profile and average slope (in degrees) of T1. c) Elevation profile and average slope (in degrees) of T2. d) Elevation profile and average slope (in degrees) of T3.


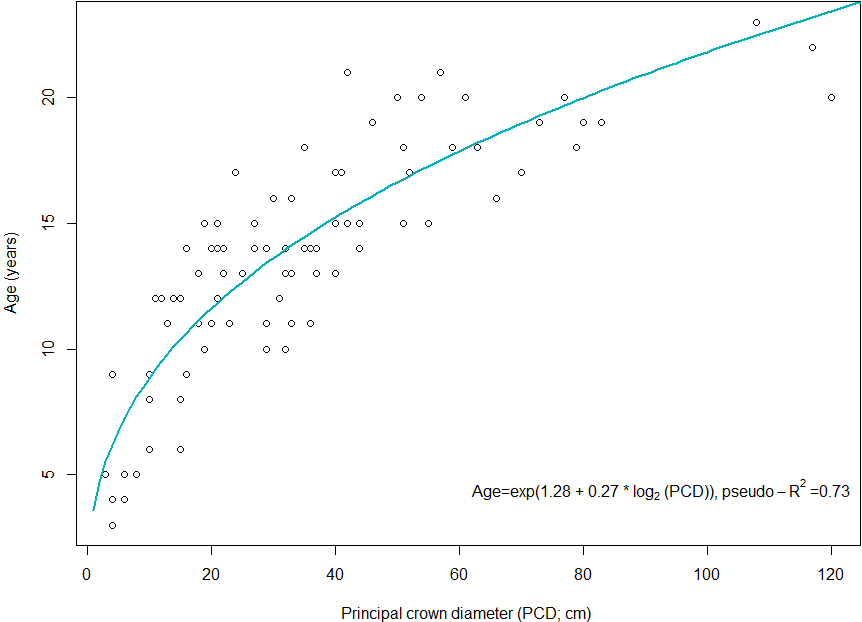


Figure S3*:* Allometric relationship between individual size (represented by the principal crown diameter (PCD)) and age of *Empetrum* individuals in the allometric dataset (n=87). Line = model fit (Poisson GLM, model formula in figure).


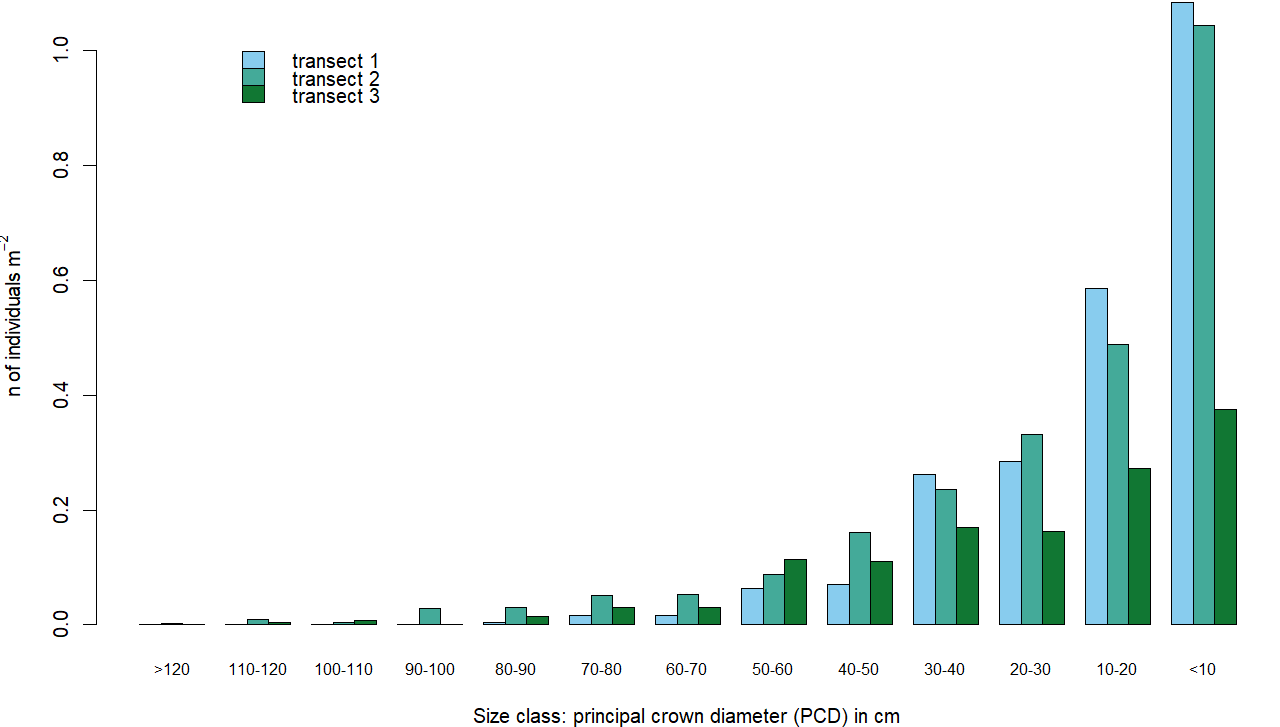


Figure S4: Size structure of *Empetrum* in transects 1–3. Size structure expressed as the average density (i.e., number of individuals per size class per m^2^) of thirteen size classes based on the principal crown diameter (PCD; classes by 10 cm). Size classes are sorted in a descending order (i.e. from the largest to the smallest PCD) to facilitate comparison with Figure 2 a).

**APPENDIX: TABLES**

Table S1: Overview of *Empetrum* individuals in transects 1–3 included in the establishment dataset. For details, see Appendix S1.

| **group** | **original n in transects 1-3** | **n lost by equipment malfunction** | **n lost by planar projection in GIS** | **analysed n in transects 1**–**3 (establishment dataset)** |
| --- | --- | --- | --- | --- |
| 2015-individuals | 30 | 0 | 2 | 28 |
| 2016-individuals | 2205 | 12 | 30 | 2163 |
| 2016-seedlings | 62 | 0 | 2 | 60 |
| ∑ | 2297 | 12 | 34 | 2251 |

Table S2: Comparison of allometric models of *Empetrum* by their AIC and BIC-values. Model 0 = null model. Model 1a = a single-predictor model with log-transformed principal crown diameter (PCD) as predictor. Model 1b = a single-predictor model with log-transformed largest diameter perpendicular to the principal (LPD) as predictor. Model 2 (a two-predictor model with log-transformed PCD and LPD; discarded for multicollinearity) added for comparison.

|  | **AIC** | **BIC** |
| --- | --- | --- |
| Model 0 | 507.34 | 512.27 |
| Model 1a | 388.68 | 396.07 |
| Model 1b | 389.82 | 397.21 |
| Model 2 | 387.40 | 397.27 |

**APPENDIX: SUPPLEMENTARY INFORMATION**

Appendix S1: Details of establishment dataset

In July 2016, we sampled, i.e. photographed, measured and mapped, 2205 *Empetrum* individuals in transects 1–3. We used GNSS survey equipment for mapping and CPOS service of Norwegian Mapping Authority (Kartverket) with stated precision < 1 cm for spatial reference. Sampled individuals with PCD below 3 cm, the size of the smallest individuals in allometry dataset, were measured from field photographs using ImageJ (Schneider et al. 2012). To avoid damage from GNSS survey equipment and ensure non-destructive sampling, we did not measure and map seedlings, i.e. small unbranched individuals (PCD ~1 cm), located in the transects within 20 cm from a larger sampled individual (n=62) in the field. Instead, we noted number, direction, and distance of seedlings to the closest sampled individual, and obtained seedling positions in ArcGIS (ESRI 2011) from field photographs and distance and direction data. All seedlings located within the transects more than 20 cm from a sampled individual were mapped directly in the field and included in n=2205. In total, establishment dataset was supposed to contain 2297 *Empetrum* individuals: 30 individuals from the allometry dataset collected in 2015 within boundaries of transects 2 and 3, 2205 individuals sampled in 2016, and 62 associated seedlings (Table A1).

Some individuals, however, had to be omitted. After sampling, we extracted mapped positions from the GNSS field controller, and due to equipment malfunction, positions of 41 individuals in three segments in transects 2 and 3 were lost. Using field and aerial photographs and ArcGIS, we recovered all lost positions of individuals in transect 2 and one segment in transect 3 (n=29). The remaining segment in transect 3 with 12 *Empetrum* individuals with unrecovered positions was omitted from further analyses (Figure A1). We also omitted 34 individuals placed outside of transect boundaries by planar projection in GIS. The establishment dataset used in further analyses thus comprised 2251 *Empetrum* individuals (Tab. A1).

Appendix S2: Details of allometric modelling

Prior to allometric modelling of age of *Empetrum* individuals based on size, we assessed correlation between the candidate predictors: the principal crown diameter (PCD) and the largest diameter perpendicular to the principal (LPD). The correlation between PCD and LPD was substantial (Kendall’s τ=0.87), and we therefore decided to use a single-predictor model to avoid multicollinearity problems (Graham 2003).

Allometric model of age with log-transformed PCD as predictor (model 1a, Table A2) performed better than the model with log-transformed LPD as predictor (model 1b). For comparison, we also built an allometric model with both log-transformed PCD and log-transformed LPD as predictors (model 2). Model 2 performed marginally better than model 1a (but not significantly; F-test p=0.08) and had lower AIC but higher BIC than model 1a. Due to the multicollinearity problem in model 2, we decided to use the single-predictor model 1a to model age of *Empetrum* individuals (Figure A3).

Appendix S3: Details of spatial point pattern analyses

We used ‘spatstat’-package (Baddeley et al. 2015) in R (R Core Team 2015) for all analyses of spatial point patterns. Positions of *Empetrum* individuals in the establishment dataset were treated as a marked point pattern with individual age as marks. Analyses of spatial point patterns required definition of an observation window (Baddeley et al. 2015), in our case transects 1–3.

To assess the effect of distance to seed sources on individual density, we used a nonparametric estimate of intensity (i.e. density of *Empetrum* individuals) as a function of covariate (i.e. distance to the nearest spoil heap edge) employed in function ‘rhohat’. To assess interactions between all *Empetrum* individuals, we used univariate inhomogeneous pair correlation function (PCF), employed in function ‘pcfinhom’. To assess potential interaction shifts over time between recruits and established individuals, we used bivariate inhomogeneous PCFs employed in function ‘pcfcross.inhom’, treating “recruits” and “established” as two categorical mark values.

For selection of smoothing bandwidth for kernel estimation of density used in specifying null models for both univariate (all individuals in the establishment dataset) and bivariate (subsets of established individuals and recruits) inhomogeneous PCFs, we used Scott’s rule of thumb employed in function ‘bw.scott’ (Scott 2015). To test deviations of observed univariate and bivariate PCFs from their respective null models, we used Diggle-Cressie-Loosmore-Ford (DCLF) test employed in function ‘dclf.test’ (Baddeley et al. 2014).

Appendix S4: Details of Figures 3–6

In Figure 3, we used pixel resolution of 0.1 × 0.1 m for individual densities and smoothing bandwidth of 1.235 m for kernel estimation. Colours for density classification (n=25 classes) were based on Jenks’ natural breaks optimisation (Jenks & Caspall 1971). We used background orthophoto from Norge i bilder (Statens kartverk et al. 2020). In Figure 4, we used actual smoothing bandwidth of 1.8056 m.

In Figure 5, we used global envelopes obtained from 78 realisations of the null model specified as an inhomogeneous Poisson process with no correlation between the points (i.e., simulated positions of *Empetrum* individuals with the same density as observed in transects 1–3).

In Figure 6, we used global envelopes obtained from 78 realisations of the null model specified as an inhomogeneous multitype Poisson process with no correlation between the points (i.e., simulated positions of *Empetrum* recruits relative to the simulated positions of established individuals) for each time-point. Densities of established individuals and recruits varied over time, and null models were therefore time-point-specific.

REFERENCES

Baddeley, A., Diggle, P. J., Hardegen, A., Lawrence, T., Milne, R. K., & Nair, G. 2014. On tests of spatial pattern based on simulation envelopes. *Ecological Monographs, 84*(3), 477-489. https://doi.org/10.1890/13-2042.1

Baddeley, A., Rubak, E., & Turner, R. 2015. *Spatial point patterns: methodology and applications with R*. New York: Chapman and Hall/CRC.

ESRI, R. 2011. ArcGIS desktop: release 10. *Environmental Systems Research Institute, CA*.

Graham, M. H. 2003. Confronting multicollinearity in ecological multiple regression. *Ecology, 84*(11), 2809-2815. https://doi.org/https://doi.org/10.1890/02-3114

Jenks, G. F., & Caspall, F. C. 1971. Error on choroplethic maps: definition, measurement, reduction. *Annals of the Association of American Geographers, 61*(2), 217-244. Retrieved from: https://www.tandfonline.com/doi/abs/10.1111/j.1467-8306.1971.tb00779.x

Kartverket Bergen. 2014. Sogndal_Aurland_Lærdal 2014. Retrieved from: https://hoydedata.no/LaserInnsyn/. https://hoydedata.no/LaserInnsyn/

R Core Team. (2015). R: A language and environment for statistical computing. Vienna, Austria: R Foundation for Statistical Computing. Retrieved from http://www.R-project.org/

Scott, D. W. 2015. *Multivariate density estimation: theory, practice, and visualization*: John Wiley & Sons.

Schneider, C. A., Rasband, W. S., & Eliceiri, K. W. 2012. NIH Image to ImageJ: 25 years of image analysis. *Nature Methods, 9*(7), 671-675. https://doi.org/10.1038/nmeth.2089

Statens kartverk, Geovekst, & kommunene. 2020. Aurland 2019. Retrieved from: https://norgeibilder.no/. Retrieved 1.12.2020 https://norgeibilder.no/
